# RETRACTION: Traditional Chinese Medicine Qingre Huoxue Decoction Enhances Wound Healing in Through Modulation of Angiogenic and Inflammatory Pathways

**DOI:** 10.1111/iwj.70616

**Published:** 2025-04-21

## Abstract

**RETRACTION:** LiW. , ChenY. , CaiZ. , HeX. , YangL. , ZhuJ. , and WangW. , “Traditional Chinese Medicine Qingre Huoxue Decoction Enhances Wound Healing in Through Modulation of Angiogenic and Inflammatory Pathways,” International Wound Journal 21, no. 3 (2024): e14724, 10.1111/iwj.14724.38439195 PMC10912365

The above article, published online on 04 March 2024, in Wiley Online Library (http://onlinelibrary.wiley.com/), has been retracted by agreement between the journal Editor in Chief, Professor Keith Harding; and John Wiley & Sons Ltd. Following an investigation by the publisher, all parties have concluded that this article was accepted solely on the basis of a compromised peer review process. In addition, further investigation by the publisher found that some citations were irrelevant and did not adequately support the conclusions presented in this article. The investigation also found an inconsistency between the dates of sample collection and euthanasia of research subjects. In view of the clear evidence of compromised peer review, the parties agreed that the paper must be retracted. The authors did not respond to our notice regarding the retraction.

**RETRACTION:** W. Li , Y. Chen , Z. Cai , X. He , L. Yang , J. Zhu , and W. Wang , “Traditional Chinese Medicine Qingre Huoxue Decoction Enhances Wound Healing in Through Modulation of Angiogenic and Inflammatory Pathways,” International Wound Journal 21, no. 3 (2024): e14724, 10.1111/iwj.14724.38439195 PMC10912365

The above article, published online on 04 March 2024, in Wiley Online Library (http://onlinelibrary.wiley.com/), has been retracted by agreement between the journal Editor in Chief, Professor Keith Harding; and John Wiley & Sons Ltd. Following an investigation by the publisher, all parties have concluded that this article was accepted solely on the basis of a compromised peer review process. In addition, further investigation by the publisher found that some citations were irrelevant and did not adequately support the conclusions presented in this article. The investigation also found an inconsistency between the dates of sample collection and euthanasia of research subjects. In view of the clear evidence of compromised peer review, the parties agreed that the paper must be retracted. The authors did not respond to our notice regarding the retraction.

